# Editorial: RNA-Protein Interactions in mRNA Translation and Decay

**DOI:** 10.3389/fmolb.2021.803063

**Published:** 2021-11-23

**Authors:** Purusharth I. Rajyaguru, Stephan Vagner, Elena Evguenieva-Hackenberg

**Affiliations:** ^1^ Department of Biochemistry, Indian Institute of Science (IISc), Bangalore, India; ^2^ PSL Research University, Institut Curie, Paris, France; ^3^ Institute of Microbiology and Molecular Biology, University of Giessen, Giessen, Germany

**Keywords:** posttranscriptional gene control, RNA granules, translation, mRNA decay, RNA-binding proteins

mRNA fate is determined by its protein partners. Association of mRNAs with RNA-binding proteins (RBPs) in the cytoplasm regulates their fate from their translation, storage to decay. Identification of novel RBPs and understanding the relevance of known mRNA-protein interactions still remains an exciting thrust area of RNA biology. Understanding how physiological cues experienced by the cell lead to changes in RNA-protein interactions, eventually leading to altered proteome, is an active field of research. RNA-protein complexes assembled in RNA granules (P-bodies and stress granules) play a key role in determining mRNA fate.

The aim of our topic was to highlight studies focusing on above aspect of RNA Biology. This has been accomplished through eight articles focusing on different aspects of RNA-protein interactions.

The role of RBPs in disease-related topics has been addressed in three articles. Behari et al., report the conserved RNA-binding activity of Inositol-5-phosphate-4-kinase (PIP4K2A) which was reported earlier in *Plasmodium* and further confirmed in *Drosophila* and *Toxoplasmi*. Interestingly the RNA-binding activity is independent of its kinase activity. The authors propose that the RNA-binding activity of PIP4K2A may be important for posttranscriptional gene control in *Plasmodium* propagation as well as in multicellular host organism. Further, in the context of solid tumors, Glaß et al., identify three mRNA targets of oncofetal IGF2 mRNA-binding protein (IGF2BP1). These transcripts are stabilized by IGF2BP1 and are involved in processes associated with hallmarks of cancer. Finally, de Vries et al., identify the role of P23 protein in macrophage inflammation response. P23 is a Hsp90 co-chaperone that demonstrates poly(A) RNA-binding activity. P23 contributes towards macrophage migration and phagocytic activity by stabilizing Kif15, a motor protein belonging to the kinesin family.

Two review articles in this topic provide insights into RNA granule assembly and function. Fernandez and Buchan review the scaffolding role of RNAs in the functioning of mRNPs in both the cytoplasm and the nucleus. An exciting aspect of this discussion is the emerging role of mRNAs as decoys in regulating mRNP assembly dynamics. Tweedie and Nissan highlight the role of stress granules (SGs) in response to infections by bacteria, fungi and protozoa. The assembly of SGs could affect host immune response and alter the outcome of microbial infection.

The role of RBPs in non-coding RNA biology has been addressed by two articles. Paturi and Deshmukh review the role of Dicer in the RNA-induced silencing complex (RISC) by orchestrating complex protein interaction network. Based on the observation that there is more than one distinct Dicer paralog in insects and plants as compared to single paralog in other eukaryotic organism, they discuss the role of Dicer in RNAi and other defense processes. Qin et al., on the other hand identify the role of miR-490-3p in silencing CDK1 in a liquid-liquid phase separation (LLPS) dependent manner in colon cancer cell lines.

Finally, Bheemireddy et al. address RNA-protein interactions during translation initiation using computational methods. The authors inspect the interaction of 30S ribosome subunit with mRNAs and identify ribosomal proteins S13, S19, and S20 as sensors that respond to changes induced by the interactions of the 30S subunit with the mRNA.

Overall, the collection of articles in this topic advances our understanding of the role of different RNA-binding proteins in health and disease.

